# Long-term vaccine efficacy of a 2-dose varicella vaccine in China from 2011 to 2021: A retrospective observational study

**DOI:** 10.3389/fpubh.2022.1039537

**Published:** 2022-11-08

**Authors:** Mingming Shu, Dandan Zhang, Rui Ma, Tianchi Yang, Xingqiang Pan

**Affiliations:** ^1^Ningbo Women and Children's Hospital, Ningbo, China; ^2^Ningbo Municipal Center for Disease Control and Prevention, Ningbo, China

**Keywords:** varicella, vaccine effectiveness, varicella vaccine, epidemiology, vaccine immunization strategy

## Abstract

**Objective:**

A 2-dose varicella vaccine immunization strategy has been implemented in many cities in China, but there is few evidence on a long-term evaluation of the efficacy of the 2-dose varicella vaccine from China. This study aims to assess the long-term vaccine efficacy of the two doses varicella vaccine and analysis of its influencing factors.

**Methods:**

A retrospective study was carried out in 837,144 children born between 2011 and 2017 in Ningbo, Easten China. The logistic regression was performed to estimate varicella vaccine effectiveness (VE).

**Results:**

The overall VE of 2 doses of varicella vaccine compared without the vaccine was 90.31% (89.24–91.26%), and the overall incremental VE of 2 doses of varicella vaccine compared to the 1-dose was 64.71% (59.92–68.93%). Moreover, the varicella vaccination age of the second dose and the interval between 2 doses were both associated with VE. The VE compared to that without the vaccine in children vaccinated at <4 years old was 91.22% (95%CI: 90.16–92.17%) which was higher than in children vaccinated at ≥4 years old (VE: 86.79%; 95%CI: 84.52–88.73). And the effectiveness of the vaccine was 93.60% (95%CI: 92.19–94.75%) in children with the interval of the 2 doses ≤ 24 months significantly higher than in children with the interval of ≥36 months (VE: 85.62%, 95%CI: 82.89–87.91%).

**Conclusions:**

This study provides evidence for long-term VE of the 2-dose varicella vaccine and the better age for 2-dose vaccination and the interval between 2 doses of the vaccine in China.

## Introduction

Varicella is an acute and highly contagious respiratory disease caused by the varicella-zoster virus (VZV), and is characterized by a generalized pruritic vesicular rash that usually resolves within a week, severe complications can occur in a few cases (1). According to the World Health Organization, the annual worldwide burden of varicella is assessed to be roughly 140 million cases with 4,200,000 severe complications that require hospitalization, and 4,200 deaths (2). In the absence of the varicella vaccine (VarV), about 39, 9 per 100,000 cases of varicella were hospitalized and 0.41 per million deaths were fatal, in the United States (3, 4). Currently, the incidence of chickenpox reported remains high, annual incidence rates from 300 to 1,291 per 100,000 population in Europe (5). In China, although varicella is not a notifiable infectious disease and has not been monitored and managed in a systematic way in most provinces, the literature shows that the reported incidence of chickenpox was still high (6, 7) compared with some legally notifiable infectious disease such as measles, rubella, and so on.

The VarV, developed in Japan in 1974, is the most practical and reliable way to prevent and control varicella (8). The prevalence and mortality of varicella as well as related medical care costs have been substantially decreased in the United States since a comprehensive varicella vaccination program was implemented in 1995 (9). In 1997 in China, VarV was introduced to inhibit varicella and was available in Ningbo with the immunization schedule of 1-dose for children over 1 year old since 1999. Consequently, significant reductions were observed in the number of varicella cases, outbreaks, varicella-related hospitalizations and deaths (10–12). However, due to both primary vaccine failure and decreasing vaccine-induced antibodies, many varicella breakthrough cases (1) were reported by the hospital, and outbreaks of varicella still existed in schools and kindergartens (13, 14). Hence, a 2-dose vaccination programme was suggested to protect children from developing varicella and control outbreaks in the USA in 2006 (15). Then it was recommended for children at 1 and 3 years old in Ningbo, eastern China since 2014. Whereas, the second dose immunization schedule varied: at 4–6 years old in the USA (16); at 4 years old in Beijing (17) and Hangzhou (6), China; at 4–5 years old in Qingdao, China; at 5–6 years old in most countries in Europe (18). Moreover, VarV has been adopted in routine vaccination programs for children in many countries and regions, including the United States, Australia, and Germany (19–21), but VarV was not counted in Expanded Program on Immunization (EPI) in China. Recently, some Chinese cities, involving Shanghai, Tianjin, and Suzhou, have taken the vaccine to the local EPI and are providing free vaccination for children (17, 22).

Our previous studies and other studies based on case–control study have confirmed that the effectiveness of 2-dose VarV was more effective than the 1-dose in China (13, 17, 23). However, these studies were based on short-term observation for vaccine evaluation, which may be affected by the waning vaccine-induced immunity in the future (24–26). So, it remains unclear whether 2-doses of VarV are long-term effective, as shown in Fu's study (7). In addition, the sample sizes of some of those studies were usually insufficient and the results were not consistently the same. For example, a matched case-control study by Hu et al. showed that the VE of 2-dose vaccination was 81.6% with 509 varicella cases and 1,527 controls (23), while the VE reported by Xu et al. was 98.0%, also based on a matched case-control study among 218 varicella cases and 218 matched controls (6). Besides, varicella immunization strategies may also affect the efficacy of 2-dose VarV such as the time interval between the 2 doses, the age of the recipient, etc.

Therefore, this work aims to evaluate the long-term efficacy of 2-dose VarV based on large sample sizes in Ningbo, eastern China; to study the effect of the 2-dose vaccination time interval and the 2-dose vaccination age on the efficacy of the VarV.

## Methods

### Setting and population

Ningbo is one of the 15 subprovincial cities in China and is one of the 5 separate state-planning cities in China, with a population of about 9,400,000 in 10 districts. Ningbo's Immunization Information System (IIS) was developed by the Ningbo Center for Disease Control and Prevention in 2004. When a child receives a vaccine, the vaccination is recorded not only in the child's vaccination manual, but also in Ningbo IIS. Hence, the Ningbo IIS data included the date of vaccination and the basic information of the vaccinee. Through the IIS, we selected consecutive 7-year birth cohorts from 2011 to 2017 as the target population for the study.

### Data sources and collection

We obtained basic data of the birth cohorts from 2011 to 2017 and their information on varicella, diphtheria-tetanus-pertussis (DTP), and Meningitis A C vaccinations by Ningbo IIS, including the name, birth date, gender, and current address of the vaccinee, and the date dose of vaccination. To reduce the impact of child mobility on the findings, these subjects in the birth cohorts who were older than 6 years and were not vaccinated against DTP or meningitis A C were excluded from the study. Moreover, those children with previous history of varicella disease before varicella vaccination were also omitted from the analysis.

From 2009 to 2013, the active surveillance of varicella was implemented in three districts in Ningbo which was described in the previous study (13). And since 2012, all practitioners in medical organizations in Ningbo have been required to report varicella cases by China Information System for Disease Control and Prevention (CISDCP) within 24 h, once the diagnosis of varicella was clear. Confirmed cases were clinically diagnosed according to the patient's clinical symptoms with acute onset of generalized maculopapular or vesicular rash without other known causes (27). The basic information and the disease onset data of varicella case with disease onset between January 1, 2011 and December 31, 2021 were extracted from CISDCP. Finally, we assembled a population-based cohort of VarVs by linking the above vaccination data and the chickenpox disease information.

### Varicella vaccination coverage, breakthrough and vaccine effectiveness (VE)

Between 2011 and 2021, there were five brand VarVs available in Ningbo per year, which have similar concentrations of Oka strain VZV and have the same temperature requirement (2–8°C) for cold-chain storage and transportation, as described in a previous study (13).

Vaccine coverage rate was defined as the proportion of the actual number of people vaccinated to the number of people who should be vaccinated. The annual prevalence rate of varicella is defined as the number of cases of varicella divided by the total population of the birth cohort. The breakthrough varicella was defined as a case that develops more than 42 days after vaccination, without other apparent cause (28). The breakthrough varicella infection rate (BVR) was defined as the percentage of breakthrough infections in children who received vaccines against varicella.

VE represents a percentage reduction in the incidence of diseases caused by vaccination and plays an important role in measuring the efficacy of the vaccine. It is calculated using the following equation: VE = (1-relative risk [RR]) / 100%.

### Statistical analysis

The 1-dose and 2-dose varicella vaccination coverage rate per 100, the varicella rate per 1,000 and the breakthrough infection rate per 1,000 per year were calculated for the overall cohort and stratified by birth cohort. The total breakthrough infection rate per 1,000 person years was also calculated by the life-table method for the only 1-dose vaccine and the 2-dose vaccine among all birth cohorts. The chi-square test was used to compare the coverage of the VarV, and the variance analysis was used to compare the ages between birthgroups. Logistic regression was used to calculate RRs and 95% confidence intervals (CIs). Further, the VE of 1-dose and 2-dose, and the incremental VE of 2-dose were calculated for the total cohort and stratified by birth cohort. Similarly, the VE of 2 doses was calculated stratified by vaccination age and vaccination interval.

All statistical analysis was conducted using Python language (Version 3.9.2) and the “statsmodel” package was used for the logistic regression. A *p* < 0.05 was considered as statistically significant.

## Results

### Characteristics of the subjects and vaccination rate

This study analyzed a total of 837,144 children in the birth cohort 2011–2017 with 444,272 males and 392,872 females (M/F ratio: 1.13). Table 1 shows the mean age of vaccination, the coverage of VarV and the average interval between the two doses. The VarV coverage was significantly different in each birth groups (only one dose: *x*^2^ = 284.91, *P* < 0.001; two doses: *x*^2^ = 6463.27, *P* < 0.001; at least one dose: *x*^2^ = 4389.99, *P* < 0.001). And the time interval between the doses was significant differences between all birth cohorts (*F* = 12333.84, *P* < 0.001).

**Table 1:** Characteristics of the subjects and vaccination rate.

**Birth cohort**	***N***	**Male** **(%)**	**1-dose**		**2-dose**			**≥1-dose**
			**Average age (years)**	**Coverage rate of only 1-dose (%)**	**Average age (years)**	**Average interval between the doses (months)**	**Coverage rate (%)**	**Coverage rate of ≥1-dose (%)**
2011	104,517	54.10	1.39 ± 1.01	12.36	4.19 ± 1.14	34.81 ± 14.19	83.97	96.33
2012	109,542	53.77	1.31 ± 0.9	9.82	3.64 ± 1.04	29.03 ± 12.27	87.24	97.06
2013	97,393	53.60	1.3 ± 0.84	8.08	3.53 ± 0.93	27.78 ± 10.95	89.38	97.46
2014	101,010	53.40	1.26 ± 0.72	6.13	3.4 ± 0.76	26.32 ± 9.18	91.60	97.73
2015	121,552	52.88	1.27 ± 0.62	15.99	3.32 ± 0.55	25.48 ± 7.38	72.64	88.63
2016	158,010	52.21	1.22 ± 0.5	19.43	3.28 ± 0.43	25.49 ± 6.29	66.50	85.93
2017	145,120	52.32	1.18 ± 0.37	23.02	3.12 ± 0.18	24.02 ± 3.86	62.51	85.53
Total	837,144	53.07	1.27 ± 0.72	14.49	3.49 ± 0.85	27.50 ± 10.22	77.29	91.77

### Varicella infection rate

**Figure 1:**
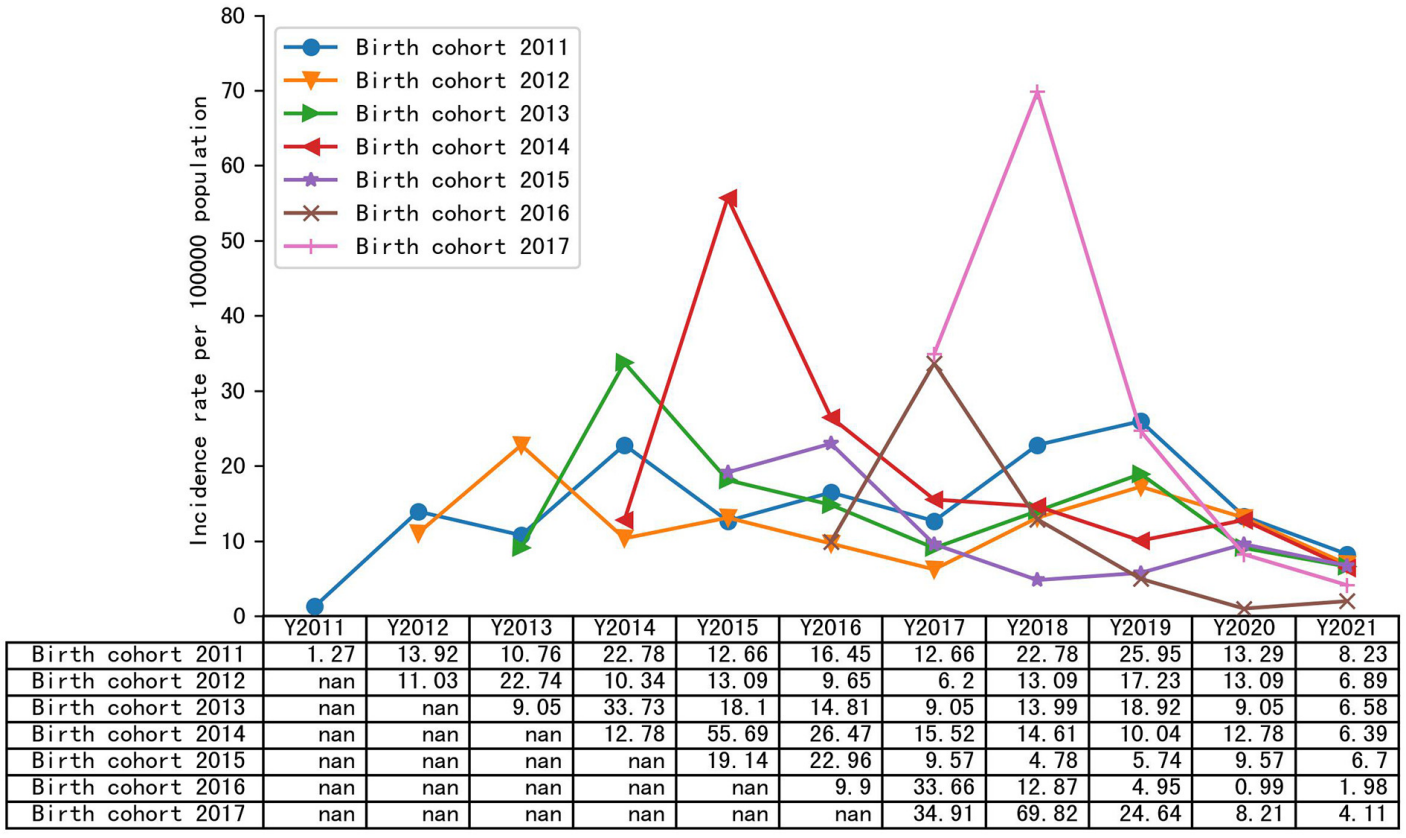
Annual infection rate of different birth cohorts from 2011 to 2021.

Figure 1 summarizes the annual incidence of varicella infection in each year among the different birth cohorts. In the 2011–2017 birth cohorts, the cumulative incidence of varicella was 292.39/100,000, 234.29/100,000, 234.47/100,000, 260.17/100,000, 177.0/100,000, 172.26/100,000, and 252.58/100,000, respectively. Except for the birth cohort 2011, the annual incidence of the remaining birth cohorts showed an upward trend within the age of 1 year and a significant downward trend after the age of 1 year. After 2020, the annual incidence of varicella in all birth cohorts declined, and in 2021, the incidence of varicella in birth cohort 2011–2017 was 12.66/100,000, 9.65/100,000, 13.99/100,000, 15.52/100,000, 10.52/100,000, 7.92/100,000, and 9.24/100,000, respectively.

### The breakthrough infection rate and the vaccine effectiveness

The total breakthrough rate was 1.95 cases per 1,000 person years (95%CI: 0.55–0.68) and 0.61 cases per 1,000 person years (95%CI: 0.55–0.68) for the only 1-dose vaccine and the 2-dose vaccine in all birth cohorts, respectively. Table 2 shows the breakthrough infection rate and the VE among the 2011–2017 birth cohorts between 2011 and 2021. In each birth cohort, children vaccinated with one dose had a significantly higher percentage of varicella infections than children vaccinated with two doses (*P* < 0.001). Compared to no vaccine the total VE was 72.53% (95% CI: 68.85–75.78%) for 1-dose and 90.31% (95% CI: 89.24–91.26%) for 2 doses.

**Table 2:** The breakthrough infection rate and the effectiveness of the vaccine.

**Birth cohort**	**Vaccination status**	***N***	**No. of cases**	**No. of breakthrough cases**	**Breakthrough infection rates (1/1,000)**	**VE (95%CI)**	***P***
2011	Unvaccinated	3,834	118			1.0	
	1-dose	12,916	110	102	7.9	74.92 (67.22–80.80)	< 0.001
	2-dose	87,767	234	228	2.6	91.80 (89.73–93.45)	< 0.001
	Difference/Incremental				5.3	67.30 (58.67–74.13)	< 0.001
2012	Unvaccinated	3,221	121				
	1-dose	10,760	68	59	5.48	85.86 (80.65–89.67)	< 0.001
	2-dose	95,561	151	131	1.37	96.48 (95.48–97.26)	< 0.001
	Difference/Incremental				4.11	75.12 (66.14–81.71)	< 0.001
2013	Unvaccinated	2,475	89				
	1-dose	7,867	74	65	8.26	77.64 (69.11–83.81)	< 0.001
	2-dose	87,051	122	111	1.28	96.58 (95.46–97.42)	< 0.001
	Difference/Incremental				6.98	84.69 (79.19–88.74)	< 0.001
2014	Unvaccinated	2,294	108				
	1-dose	6,189	61	53	8.56	82.49 (75.59–87.44)	< 0.001
	2-dose	92,527	116	97	1.05	97.88 (97.20–98.39)	< 0.001
	Difference/Incremental				7.51	87.86 (83.02–91.33)	< 0.001
2015	Unvaccinated	13,820	73				
	1-dose	19,439	40	33	1.7	67.97 (51.65–78.78)	< 0.001
	2-dose	88,293	72	59	0.67	87.41 (82.24–91.07)	< 0.001
	Difference/Incremental				1.03	60.69 (39.79–74.33)	< 0.001
2016	Unvaccinated	22,237	93				
	1-dose	30,694	34	22	0.72	82.91 (72.80–89.27)	< 0.001
	2-dose	105,079	47	32	0.3	92.75 (89.16–95.15)	< 0.001
	Difference/Incremental				0.42	57.54 (26.92–75.33)	< 0.001
2017	Unvaccinated	20,996	143				
	1-dose	33,411	47	29	0.87	87.32 (81.10–91.50)	< 0.001
	2-dose	90,713	56	27	0.3	95.66 (93.45–97.12)	< 0.001
	Difference/Incremental				0.57	65.74 (42.12–79.72)	< 0.001
Total	Unvaccinated	68,877	745				
	1-dose	121,276	434	363	2.99	72.53 (68.85–75.78)	< 0.001
	2-dose	646,991	798	686	1.06	90.31 (89.24–91.26)	< 0.001
	Difference/Incremental				1.93	64.71 (59.92–68.93)	< 0.001

### The risk factors for the two-dose VE

Table 3 shows the VarV vaccination age of the second dose and the interval between 2 doses were all associated with the VE. The breakthrough infection rate in children vaccinated at <4 years old was 0.96/1,000 which was significantly lower than in children vaccinated at ≥4 years old with *P* < 0.001. And the VE compared to that without the vaccine in children vaccinated at <4 years old was 91.22% (95%CI: 90.16–92.17%) which was higher than in children vaccinated at ≥4 years old (VE: 86.79%; 95%CI: 84.52–88.73). And the incremental VE in children vaccinated at <4 years old was 68.05% (95%CI: 63.40–72.10%) which was also higher than in children vaccinated at ≥4 years old (VE: 51.92%; 95%CI: 42.73–59.63%), compared to the vaccine with one dose. Further hierarchical analysis also showed the same results (Table 3).

**Table 3:** The varicella vaccine effectiveness stratified by the vaccination age and the interval time between two doses.

**The second dose vaccination age (Years old)**	**The interval between 2 doses (Months)**	***N***	**No. of breakthrough cases**	**Breakthrough infection rates (1/1,000)**		***P***	**Unvaccinated as reference**			**1-dose vaccination as a reference**		
							**VE**	**95%CI**	***P***	**VE**	**95%CI**	***P***
Total	≤ 24	16,1518	113	0.70	44.02	< 0.001	93.60	92.19–94.75	< 0.001	76.69	71.20–81.13	< 0.001
	25–36	387,405	418	1.08			90.12	88.86–91.24	< 0.001	64.04	58.60–68.76	< 0.001
	>36	98,068	154	1.57			85.62	82.89–87.91	< 0.001	47.64	36.76–56.64	< 0.001
< 4	≤ 24	146,028	105	0.72	12.23	< 0.001	93.42	91.92–94.64	< 0.001	76.04	70.22–80.72	< 0.001
	>24	367,125	387	1.05			90.35	89.08–91.47	< 0.001	64.87	59.45–69.56	< 0.001
	Total	513,153	492	0.96			91.22	90.16–92.17	< 0.001	68.05	63.40–72.10	< 0.001
≥4	≤ 24	15,490	8	0.52	10.52	< 0.001	95.27	90.51–97.64	< 0.001	82.79	65.32–91.46	< 0.001
	25–36	20,926	31	1.48			86.43	80.55–90.53	< 0.001	50.60	28.69–65.77	< 0.001
	>36	97,422	154	1.58			85.52	82.77–87.83	< 0.001	47.29	36.34–56.35	< 0.001
	Total	133,838	193	1.44			86.79	84.52–88.73	< 0.001	51.92	42.73–59.63	< 0.001

## Discussion

Previous studies have confirmed that the effectiveness of the two-dose VarV in China was higher than that of the one-dose (13, 17, 23). However, these studies were either short-term observations for evaluation or were based on a small sample size of case-control studies. In this study, we observed the birth cohorts from 2011 to 2017 between 2011 and 2021 in Ningbo to fill in the missing data on the long-term VE and its influence factors in China.

This study showed that the total breakthrough infection rate of the 2-doses vaccine was 0.61 cases per 1,000 person years (95%CI: 0.55–0.68/1,000 person years). Similar results were obtained in a meta-analysis by including 27 original articles and used random effects model, which showed the breakthrough of 2.2 cases per 1,000 person years (95% CI: 0.5–9.3/1,000 person years) in children vaccinated with 2 doses (29). And the breakthrough infection rate of 2-dose series showed that breakthrough infection rate of 2-doses vaccine was significantly lower than that of the 1-dose among birth cohorts between 2011 and 2017. Similar conclusions were also drawn by Hu et al. (23), which suggests that the rate of evolution of varicella may be significantly reduced by two doses of the VarV. Moreover, the breakthrough infection rate of 2-doses series in this study was 2.6 per 1,000 population for post-vaccination about 7 years and was no more than 1.37 per 1,000 population for post-vaccination <6 years, which was consistent with the breakthrough infection rate (1.2/1,000) from the previous active surveillance project for ~2 years after vaccination (13). This indicates that the 2 doses of vaccine can be effective and durable in preventing varicella breakthrough infection.

Further, many studies (20, 23, 30–32) reported that the 2-dose VE was higher than 1-dose. A recent meta-analysis (30) with 22 studies showed that the incremental VE of 2-dose vaccination was 63% (95% CI: 36–79%) in cohort studies, which was similar with that of 64.71 (59.92–68.93) in our study. And the meta-analysis also showed that 2-dose varicella vaccination produced higher levels of immunogenicity than one-dose vaccination. This means that the vaccine with two doses has improved VE compared to the vaccine with one dose. However, the VE of 2-dose was varied from 81.6 to 100% according to the different studies (13, 23, 33). The reason for this might be partially attributed to the difference in sample size, research design methods and the time after vaccination. For example, the estimate of the VE in varicella outbreaks is usually lower than that of other studies because when the infection pressure is high, the vaccine performance tends to be underestimated (34). A new meta-analysis (35) with 12 studies and 87,196 individuals also proved that the VE of outbreak studies was lower than in non-outbreak studies. It showed that the pooled two-dose VE was 90% (95% CI: 69–97%), similar to the efficacy of the vaccine of 90.31 with a population of 837,144 in this study.

Our findings also showed that 2-dose VE was increased from 91.80% among birth cohort 2011 to among birth cohort 2014, but it decreased in birth cohort 2015. The reason for this may be related to the coverage rate of 2-dose vaccination (36), and the VE increase when the vaccination rate increase. Similarly, when vaccination rates in the 2015 birth cohort fell rapidly and VE also declined rapidly. This indicates that the effect of 2-dose can be maintained for a long time when the vaccine rate is sufficiently high, which also can be demonstrated by an age-structured deterministic compartment model with data from Korea's population projection (37). Similar conclusions were drawn by the World Health Organization, who's systematic review showed that the two-dose of VarV could provide long-term protection of up to 14 years (2). On the other hand, the VE increased in the birth cohort in 2016 and 2017 with a lower coverage rate of two doses of vaccine, which may be because the immunity from the first dose vaccine did not decrease in the short term.

Moreover, one of the most striking observation to emerge from the analysis was that the age at the second vaccination and the interval between 2 doses affected the VE of 2-dose vaccine. In this study, the VE of 2 doses of varicella in vaccine recipients receiving vaccines at <4 years of age was higher than that of vaccine recipients injecting vaccines at ≥4 years of age, whether the interval between the two vaccine doses was >24 months, suggesting that the second dose of varicella could be given as soon as possible when the vaccination interval meets the basic requirements (the basic requirements of the interval was more than 3 month in Ningbo). These results are not in line with those of Black et al., whose data do not show any difference in the effectiveness of the vaccine depending on the age of the vaccination. The reason for that was the maximum age for vaccination in their study was 23 months which was not consistent with our study (38).

In addition, this study revealed that the shorter the two doses intervals, the better the effect of the vaccine, suggesting that different national or regional recommendations relating to this interval will result in different VE of the 2-dose vaccine. These findings are consistent with the previous study showing the shortening of the interval between the first dose and the second dose of vaccination should reduce breakthrough varicella and outbreaks in preschool (39). Conversely, the results do not contradict previous research by Rieck et al. which found there was no significant difference in VE of the 2-doses vaccine in all investigated time intervals >27 days up to >3 years between varicella doses (32). This may be due to the protection induced by one dose vaccine of varicella did not decrease in their study, but our study and other study revealed the reduced long-term VE of one-dose vaccination and the reduction in efficacy over time (13, 14, 40).

Our study has strengths and limitations that deserve mention. The most significant strengths of this study are the largest cohort size with high-quality detailed antenatal records from CISDCP and NBIIS, which gives larger power to the estimated VE, and community-based evaluation for long-term VE of 2-dose VarV in China. In addition, the association between the VE and influencing factors including the age of the 2-dose vaccination and the interval of two doses of the vaccines was also analyzed. Our study has limitations as well. First, given the large sample size and the difficulty of collecting sufficient specimens of maculopapular rashes (41), the diagnosis of the disease in this study was made only for clinical reasons. Nonetheless, this could lead to a certain degree of incorrect classification, which has a very limited impact on the assessment of the VE (13). Second, due to the prevention and control measures of COVID-19, all birth cohorts had lower incidence rates in 2020 and 2021, which could result in an overestimation of the vaccine efficacy. Last, it was possible that the cases could not be visited or not diagnosed in the hospital, which could also lead to a reduction in the rate of infection in the breakthrough case. Nevertheless, the quarterly report on the assessment of infectious diseases did not contain a lack of reporting on varicella diseases (13). And the high level of reporting of varicella disease using CISDCP by experienced doctors has been maintained. As a result, true VE estimates have been made as far as possible through these strengths.

## Conclusions

Our results provide compelling evidence for the long-term VE of 2-dose VarV and the better age for 2-dose vaccination and the interval between 2 doses of the vaccine, suggesting that it is very necessary to recommend a two-dose immunization strategy for controlling varicella, and policy makers should take into account the age of the 2-dose vaccination and the interval between the two doses for making varicella immunization schedule in China.

## Data availability statement

The raw data supporting the conclusions of this article will be made available by the authors, without undue reservation.

## Ethics statement

The studies involving human participants were reviewed and approved by Ningbo Municipal Center for Disease Control and Prevention Review Board. Written informed consent from the participants' legal guardian/next of kin was not required to participate in this study in accordance with the national legislation and the institutional requirements.

## Author contributions

MS and XP conceptualized and designed the study, carried out the initial analyses, drafted the initial manuscript, and reviewed and revised the manuscript. DZ and TY coordinated and supervised data collection, and critically reviewed the manuscript for important intellectual content. All authors contributed to the article and approved the submitted version.

## Funding

The research was sponsored by funding Zhejiang Province Basic Public Welfare Research Program Project (LGF20H260006), Science and Technology Planning Project of Ningbo Science and Technology Bureau (202002N3185), and Medical Health Science and Technology Project of Zhejiang Provincial Health Commission (2023KY1130).

## Conflict of interest

The authors declare that the research was conducted in the absence of any commercial or financial relationships that could be construed as a potential conflict of interest.

## Publisher's note

All claims expressed in this article are solely those of the authors and do not necessarily represent those of their affiliated organizations, or those of the publisher, the editors and the reviewers. Any product that may be evaluated in this article, or claim that may be made by its manufacturer, is not guaranteed or endorsed by the publisher.
